# Relationship between food perceptions and health-related quality of life in a prospective study with breast cancer patients undergoing chemotherapy

**DOI:** 10.6061/clinics/2018/e411

**Published:** 2018-11-16

**Authors:** Eduarda da Costa Marinho, Isis Danyelle Dias Custódio, Isabela Borges Ferreira, Cibele Aparecida Crispim, Carlos Eduardo Paiva, Yara Cristina de Paiva Maia

**Affiliations:** I Programa de Pos Graduacao em Ciencias da Saude, Universidade Federal de Uberlandia, Uberlandia, MG, BR; II Faculdade de Medicina, Universidade Federal de Uberlandia, Uberlandia, MG, BR; III Divisão Mama & Ginecologia, Departamento de Oncologia Clinica, Grupo de Pesquisa em Cuidados Paliativos e Qualidade de Vida (GPQual), Fundacao Pio XII - Hospital de Cancer de Barretos, Barretos, SP, BR

**Keywords:** Breast Neoplasms, Antineoplastic Agents, Food Consumption, Appetite, Quality of Life

## Abstract

**OBJECTIVE:**

To correlate the perceptions related to dietary intake with the domains and subscales of health-related quality of life (HRQL) in women with breast neoplasms receiving chemotherapy.

**METHODS:**

In this prospective study, 55 women with breast cancer were followed up during chemotherapy at three different times (T0, T1, T2). Before chemotherapy, perceptions related to food consumption were evaluated. HRQL was analyzed with the EORTC QLQ-C30 and Br23 instruments 21 days after each investigated cycle. The differences (T2-T0) in the subscales and HRQL domains were correlated with the differences (T2-T0) in the appetite scores. Spearman's correlation was used to verify a possible correlation between differences in functional and overall HRQL domains (T2-T0) and differences in appetite scores for certain foods and between the differences in some subscales of EORTC QLQ-C30 and Br23 (T2-T0) and differences in appetite scores for certain food groups (T2-T0).

**RESULTS:**

Correlations between pain and appetite for bitter taste and between an increased appetite for juices and pain intensification or fatigue were identified, and pain was correlated with an appetite for starchy foods. An appetite for vegetables, legumes and meat/eggs was correlated with physical function. The only significant correlation with social functions occurred between the appetite for sweet foods and these functions. We found a correlation between overall health, emotional function, social function and physical function and the appetite for juices.

**CONCLUSION:**

Chemotherapy alters the individual's relationship with food and, consequently, the individual's HRQL.

## INTRODUCTION

Breast cancer (BC) is a serious, stressful and life-threatening disease 1. However, over the years, the survival rates of patients have increased due to early diagnosis and progress in response to treatment. Disease control and general patient well-being are the aim of therapy 2.

Health-related quality of life (HRQL) is assessed by self-perceptions of physical, psychological and social well-being, and HRQL reflects the perception of the impact of BC detection and therapy on patients' daily activities 3. The HRQL assessment can provide information regarding the care of these women. Regarding nutrition and nutrient intake-related side effects, nutritional status, body composition and food consumption can play important roles in the HRQL of patients 4. In fact, healthy eating habits 5 and adequate body weight 1,4 are directly related to the HRQL of women with BC.

Food intake is affected by hunger, the pleasantness of the taste of the food in the oral cavity, food reward, food availability, social influences and other factors 6 that may favor the occurrence of symptoms of anxiety and depression, pain, nausea, vomiting, constipation, taste disturbance and xerostomia during antineoplastic treatment 7. In this context, the relationship of the individual with food may change during chemotherapy (CT); tasty preparations before treatment become distasteful or provoke malaise 8, and a hedonic alteration may occur, which means that although the food has the same taste, the taste is no longer regarded as pleasant 9.

In a systematic review that considered HRQL and dietary changes in cancer patients, Kassianos et al. 10 found studies showing significant differences between dietary changes and modifications in HRQL, while other studies did not find this difference. The authors also verified that the relationship between dietary changes and changes in HRQL exhibits the following dynamic: from dietary change (factor) to HRQL (outcome). However, some authors state that several studies are limited because of their cross-sectional research designs, which do not allow assessments of causal relationships between HRQL and changes in cancer patients' diets.

Regarding perceptions related to food intake and HRQL during CT, the literature is scarce, and this study may contribute new knowledge about the subject. In this regard, we aimed to correlate perceptions related to dietary intake in terms of the domains and subscales of HRQL with the appetite of women with BC undergoing CT. We hypothesized that polychemotherapy alters the individual's relationship with food, resulting in a decline in meal enjoyment during treatment.

## MATERIALS AND METHODS

### Clinical variables

A study conducted in 2014-2015 enrolled women with stage I-III BC undergoing CT and included three sequential assessments: principle (T0), intermediate (T1) and end of CT (T2). The timing of the evaluations varied from approximately four to six months according to the CT regimen used.

The subjects were selected in the hospital while waiting for their first oncological medical appointments prior to CT infusion. Clinical data were obtained through interviews and medical record consultations.

The evaluations were performed at three stages of treatment: on the day of the first CT cycle (T0), the day of the intermediate cycle (T1) and the day of the last cycle (T2), with the intermediate cycle varying according to the CT protocol used. When the FAC (5-Fluorouracil + Adriamycin + Cyclophosphamide) or CMF (Cyclophosphamide + Methotrexate + 5-Fluorouracil) protocols were used, the intermediate cycle (T1) was the third stage. When the AC-Docetaxel (Adriamycin + Cyclophosphamide with Docetaxel) or AC-Paclitaxel (Adriamycin + Cyclophosphamide with Paclitaxel) regimens were used, the fourth cycle was considered an intermediate cycle (T1).

### Inclusion criteria

All women with BC treated at a university hospital (Hospital de Clínicas da Universidade Federal de Uberlândia, Minas Gerais, Brasil) from August 2014 to May 2015, aged 18 years or over, were invited to participate in the study immediately before their first cycles of CT, without nutritional monitoring and with the capacity to respond to the instruments. In cases of alteration of treatment due to toxicity or disease progression, the patient was excluded from the analyses.

### Sample size

The sample size was calculated using the G*Power 3.1 software and was based on an F test repeated measures ANOVA with an effect size f of 0.25, an alpha level of 0.05, 95% power, one group of participants and three mensurations. At the final follow-up, a total sample size comprising 43 women was required, and 20% was added to the sample to account for possible losses, for a minimum of 52 women at baseline (T0).

### Variables analyzed

#### Perceptions related to food consumption

To investigate food perceptions, an instrument was used at each of the three CT stages (T0, T1 and T2), rather than infusion of chemotherapeutic agents. Previously, a pilot study of 15 women with BC receiving CT was conducted in the same institution to test the instrument.

This institution offered meals three times a day for patients awaiting medical consultation or CT. The volunteer's time in the institution was monitored, and the form for evaluating perceptions related to food consumption was used by a previously trained nutritionist. In cases where patients did not consume food during their times in the waiting room, only the motive for non-consumption was assessed, with registration according to thirteen categories, namely, “I never feed myself at this time”, “I do not feel hungry”, “There is nothing to eat”, “I do not like what there is to eat”, “I am nauseated”, “I am vomiting”, “I have a sore mouth”, “I have a dry mouth”, “I have difficulty swallowing”, “The smell bothers me”, “I am in severe pain”, “I have stomach pains”, and “I have a burning sensation in the stomach”.

Using a 0-10-cm visual analog scale (VAS) 11,12, parameters including hunger, meal enjoyment and appetite for the determined food groups were assessed. To evaluate hunger, participants were asked “How hungry were you before you ate?”, and the responses were marked in the 10-cm VAS (0 cm representing “no hunger” and 10 cm representing “very hungry”). Appetite for each determined food group was also evaluated with a 10-cm VAS (0 cm meaning “none” and 10 cm meaning “a lot”). The food groups evaluated were 1) foods rich in starch; 2) legumes; 3) vegetables; 4) meats and eggs; 5) soups, broths and scalded; 6) fruits; 7) fruit juice; 8) milk; 9) dairy products; 10) salty foods; 11) sweet foods; 12) acidic foods; 13) bitter foods; and 14) spicy foods. Individuals were instructed to respond to this question only on the basis of appetite before the meal, without thinking about the nutritional facts. The overall appetite was determined by means of the median of the other appetite values at the three stages, as proposed by Spiegel et al. 13. To evaluate meal enjoyment, the question asked after the meal was “How much did you enjoy your food?”, and the answer was marked in another 10-cm VAS (0 cm representing “not at all” and 10 cm representing “a lot” on the right).

#### Health-related quality of life

For the evaluation of HRQL, two instruments developed by the European Organization for Research and Treatment of Cancer (EORTC) were used, namely, the general QLQ-C30 and its BC module QLQ-Br23, in the Brazilian Portuguese versions, after gaining permission from the EORTC for use in scientific research. Both instruments were previously validated in the Brazilian Portuguese language 14. These forms were applied to the consultations after each of the three CT stages, namely, 21 days after T0, T1 and T2, always before CT.

#### Food preferences and aversions

Food preferences and aversions were assessed (until the saturation of responses) at two different times of the study: before the participant started CT (T0) and 21 days after the last CT cycle (T2 + 21 days).

#### Statistical analysis

GraphPad Prism^®^ 5.0 *software* was used to analyze the data. The distribution of data was obtained using the Kolmogorov-Smirnov test (K-S test). The differences between the median values of HRQL, hunger and meal enjoyment of the three evaluation times were verified using the Friedman test and Tukey's post hoc test. The proportions of food aversions and preferences between the beginning and the end of CT were assessed with Fisher's exact test. Median values of appetite for certain food groups were assessed between T0, T1 and T2 with the Kruskal-Wallis test and Dunn's post hoc test.

Spearman's correlation was used to verify a possible correlation between differences in functional and overall HRQL domains (T2-T0) and differences in appetite scores for certain foods and between the differences of some subscales of EORTC QLQ-C30 and Br23 (T2-T0) and differences in appetite scores for certain food groups (T2-T0).

#### Ethical considerations

This study was developed in accordance with the Declaration of Helsinki and the CNS 466/2012 Resolution and with the ethical standards of the institutional and/or national research committee. The research was approved by the Committee for Ethics in Research with Human of the Universidade Federal de Uberlândia (Protocol number: 721.977/14).

#### Consent to participate

All research participants signed informed consent forms.

## RESULTS

Between August 2014 and May 2015, 82 women were screened, of whom 71 were eligible. During follow-up, 16 participants were excluded due to non-adherence, death or missing data. Thus, 55 women participated in the study, and the mean age was 51.5±10.1 (29-66) years. The numbers of women screened, eligible, recruited and followed up during the study are available in the article published by Marinho et al. 15.

Clinical data are presented in Table 1. Most women were postmenopausal (n=34, 61.82%) and had ductal invasive carcinoma (n=53, 96.36%). The most prevalent type of surgery was conservative surgery (n=24; 43.64%), allowing more use of adjuvant CT (n=31; 56.36%). The most commonly used CT protocol was AC-T (n=41; 74.55%).

When evaluating the hunger scale, a median score was verified at T0, T1 and T2 (*p*=0.113), which is equivalent to moderate intensity. Meal enjoyment decreased during CT and was recovered at the end of treatment (*p*=0.021; Table 2).

At the beginning of treatment, the preferred foods were meats (36.36%), rice (29.09%) and vegetables (27.27%). After CT, the preferences changed and became fruits (10.90%), vegetables (7.27%) and salty foods (5.45%). In addition, the preference for meats (*p*<0.0001), rice (*p*<0.0001), vegetables (*p*=0.010), legumes (*p*=0.0004), sweets (*p*=0.002) and bakery products (*p*=0.027) decreased at the end of treatment. Before CT, the most reported aversions were vegetables (30%), fruits (18.18%) and meats (9.09%). At the end of treatment, the most distasteful foods were meats (27.27%), legumes (16.36%) and coffee (14.54%). In addition, aversions to vegetables decreased (*p*=0.0002), and aversions to meats (*p*=0.024) and coffee increased (*p*=0.005; Table 3).

Regarding HRQL, overall health was close to 75 points in all periods, and although there were no alterations between the assessments, physical, cognitive and role functions suffered losses from treatment (*p*<0.001). The symptoms that worsened were fatigue (*p*<0.01), nausea (*p*=0.01) and pain (*p*=0.01). Notably, nausea increased by 282% between the beginning and the end of treatment. Body image also deteriorated as CT progressed (*p*=0.04; Table 4).

Table 5 shows the correlations between differences in functional and overall HRQL domains (T2-T0) and the differences in appetite scores for certain foods (T2-T0). Overall health (Rho=-0.446), emotional function (Rho=-0.557), social function (Rho=-0.540) and physical (Rho=-0.388) function showed a negative correlation with the appetite for juices. The appetite for vegetables (Rho=-0.461), legumes (Rho=-0.538) and meats/eggs (Rho=-0.431) had a negative correlation with physical function. The only positive correlation related to social function occurred with the appetite for sweet foods (Rho=0.362).

There was also a correlation between differences in some EORTC QLQ-C30 and Br23 subscales (T2-T0) and differences in appetite scores for certain food groups (T2-T0). A positive correlation was observed between appetite for juices and intensification of pain (Rho=0.44, *p*<0.05) or fatigue (Rho=0.59, *p*<0.01). Pain was also positively correlated with the appetite for foods rich in starch (Rho=0.380, *p*<0.05). Furthermore, we observed a positive correlation between pain and appetite for bitter flavor (Rho=0.366, *p*<0.05, Table 6).

## DISCUSSION

In this study, we analyzed women with stage I-III BC undergoing polychemotherapy, and the results suggest that CT modifies the individual's relationship with food because a decline in meal enjoyment was noted during the treatment. In the analysis of food preferences and aversions, there were reductions in preference for meats, rice, vegetables, legumes, sweets and bakery products; however, aversions to coffee and meats increased, and aversions to vegetables decreased. In addition, the physical, cognitive and role functions suffered losses during treatment. Appetite for juices was negatively related to overall health and emotional, social and physical functions and positively related to the intensity of pain and fatigue. In addition, pain was directly related to the appetite for bitter and starchy foods. Social function was positively correlated with appetite for sweet foods. In this regard, this prospective study brings new knowledge to help understand the perceptions related to food consumption that interfere with the use of meals and HRQL. These findings are relevant because they complement the recent publications of our research group, which demonstrate that women with BC undergoing CT present impairments in dietary quality, thereby negatively impacting their nutritional statuses 15-17.

### Hunger and meal enjoyment

The hunger scale demonstrated moderate intensity (median=5) at the three time points; that is, the basal state (T0) was not modified by treatment. A gradual reduction in these scores was expected due to possible gastrointestinal complications resulting from treatment 7. That the hunger remained constant was assumed because hunger was evaluated before the infusion of CT at T0, T1 and T2, thus avoiding interference from acute side effects.

A qualitative study explored patients' experiences of smell and taste modifications during CT 9. Regarding meal enjoyment, smell and taste alterations caused feelings of frustration, irritability, annoyance, melancholy and sadness, and because of this, patients reported that food no longer brought a feeling of enjoyment or consolation. Following this line of reasoning, Alvarez-Camacho et al. 18 observed that greater smell and taste changes were related to decreases in social, emotional, physical and global functions, which may limit participation in social and recreational activities. Decreased motivation to eat family or favorite foods has been reported by patients undergoing CT and may be mistaken for a sensory change instead of a hedonic change.

### Food preferences and aversions

At T0, the favored foods were meats, rice and vegetables. At T2, the preferences became fruits, vegetables and salty foods. Notably, the preference for meats, rice, vegetables, legumes, sweets and bakery products decreased during the course of the treatment. In contrast to our findings, Coa et al. 19 noted that the preferred foods of women undergoing treatment for BC were soups, poultry, and fish, in this order. Verde et al. 8 found an increase in preference for orange, tangerine and papaya after the chemotherapeutic cycles, confirming our findings of fruits being the preferred foods at T2. However, these data conflict with those of Holmes et al. 20, who cite citrus fruits as foods avoided after CT. Verde et al. 8 also observed increases in the food preference scores for beef, rice, crackers, cookies, and ice cream after treatment, in contrast to our findings of reductions in the preferences for meats, rice and sweets. Notably, food preferences are related to genetic and social/cultural aspects 21 and may vary according to the population studied.

In the case of food aversions before CT, the most mentioned foods were vegetables, fruits and meats. After the last cycle of CT, the largest aversions were meats, legumes and coffee. In addition, the aversions to vegetables decreased, and the aversions to meats and coffee increased. The lower bitter (urea) threshold associated with the amino acid, peptide, and purine content of meat may elicit a bitter sensation, causing meat aversion 22. The reduction in the bitter taste threshold may also be related to coffee aversion because caffeine is considered a bitter substance 23. Attention is drawn to the fact that 10 to 78% of CT patients report a metallic taste. This may occur due to a change in the threshold for a bitter, salt, sour or sweet taste 24. The literature on this topic presents results that are not necessarily convergent. Steinbach et al. 25, in a cohort study of 69 participants with BC and 12 with gynecological cancer, found that the most distasteful foods were meats, fatty foods, fruits, chocolate, cream, coffee and cola-based soft drinks. Verde et al. 8 studied 25 women with breast neoplasms and observed that after CT, they showed aversions to coffee and to a group of beverages that included alcoholic beverages, tea, black coffee and sweeteners. In a systematic review, Boltong and Keast 26 affirmed that aversions to preferred or usually ingested foods such as a tea, coffee, juice, chocolate, citrus fruit or red meat are common. Thus, these authors corroborate our findings by mentioning meats and coffee as food aversions of CT patients. Although these aversions do not cause important nutritional deficits, the exclusion of some foods may negatively impact HRQL 20.

In the present study, the medians of the global health score were ≥75 points at T0, T1 and T2, in conformity to Michels et al. 14. The closer to 100 this score was, the better the HRQL would be. Although no accepted cut-off for “good” HRQL exists, HRQL was most likely scored satisfactorily in the three periods. Despite the favorable scores, some domains of HRQL showed negative impacts over the course of treatment. In agreement with Browall et al. 27, we observed a worsening of body image and of the physical and role functions, as well as increased fatigue, pain and nausea. In corroboration of the findings of Moro-Valdezate et al. 28, a reduction in cognitive function was noted. These authors also confirmed decreases in physical and role functions.

The differences in the appetite scores for determined food categories (T2-T0) were also correlated with differences between some HRQL domains (T2-T0). The lower the physical function was, the greater the appetite for vegetables, legumes and meats/eggs would be. Meats are good sources of proteins with high biological value; minerals, especially heme iron; and B vitamins 29. On the other hand, eggs are relatively more accessible and furnish essential fatty acids, protein, vitamins A and B12, choline, selenium and other essential nutrients at levels that are higher than or comparable to those found in other animal foods 30. Another source of reasonably priced proteins is legumes, which are also high in fiber, complex B vitamins, and minerals such as potassium, calcium, and iron 31. Meats, eggs and legumes are builder foods 32, which are essential for the synthesis and recovery of cells 33. During treatment, women may have produced higher appetite scores to these foods because of the association with muscle repair and consequent recovery of physical functions. In addition, anemia is frequent in patients undergoing antineoplastic treatment 34 and may have influenced the appetite for iron sources to restore health.

Regarding the inverse relationship between physical function and the appetite for vegetables, one study has suggested that unintentionally, women associate regulatory foods 32 with physical recovery because these foods are associated with a healthy diet 35. Vegetables have significant levels of minerals, vitamins, carbohydrates, fibers and proteins, as well as recognized functional roles 36. Individuals with cancer are susceptible to modifications of life habits and represent a portion of the population that is interested in healthier diets 10. Many studies have shown favorable changes in eating habits after BC diagnosis with an increased consumption of vegetables 37.

Humans are innately fond of sweet flavors 38, and these flavors are related to pleasure and well-being, promoting the gathering of people. Sweet flavors also induce the production of the neurotransmitter serotonin, which helps provide a sensation of happiness and brings emotional and symbolic significance because the taste represents affection 39. These factors help elucidate the positive relationship between an appetite for sweet food and social function at the end of treatment.

Natural juice is considered a fresh food that is rich in vitamins and a component of a healthy diet 40. In addition, because juice is a liquid, it may be a good option for patients with nausea, vomiting, xerostomia, lack of appetite, diarrhea, mucositis, anorexia, and fatigue, among other challenges 7. This may be an explanation for the appetite for juices being inversely related to overall health, physical function, emotional function and social function and directly related to pain and fatigue. As pain increased, the appetite for starchy foods (bread, crackers, cookies, potatoes, rice, etc.) also increased. Approximately 50% of cancer patients at any stage of the disease report pain 41. Pain is usually related to inflammation and is accompanied by other symptoms such as fatigue, lack of appetite and weakness 42. Thus, the participants may have preferred small meals, which are usually rich in carbohydrates 43. Some authors suggest a relationship between pain and bitter taste in specific areas of the brain. The palate is suggested to activate neural regions that overlap with pain, indicating that pain minimization can influence taste processing 44. Positive effects resulting from pain relief are related to subsequent stimuli, thus enlarging satisfaction with these stimuli 45. In animals, the assumption is that pain stimulates the opioid system, increasing preferences for sweet flavors and decreasing aversions to bitter flavors 44, which contributes to clarifying the finding of a direct relationship between pain and an appetite for sweet foods.

The study presents some limitations, including the measurement of taste immediately before CT. The changes between each CT infusion were not investigated. Future studies may investigate taste changes using diaries or other valid prospective instruments. Another limitation is the lack of an instrument to measure oral pain because muscositis is commonly diagnosed in BC patients receiving adjuvant CT. The observed correlations between pain scores and taste changes, for instance, would be easily understandable with a focus on oral pain. Unfortunately, pain scores from the EORTC QLQ-C30 are not specific for the oral cavity but instead may be a composite score for other sources of pain, mainly post-mastectomy pain syndrome. The unicentric nature of the study is another possible limitation because our findings may not be generalizable to other countries and cultures. Finally, an observation made by Kassianos et al. 10 is worth noting: researching cancer survivors rather than patients under treatment is important because HRQL can be modified as a result of the side effects of oncological treatment.

Although general health remained constant during treatment, there were negative impacts of CT on physical, cognitive and role functions. We also noticed that the appetite for certain food groups correlated with HRQL scores. The relationship between the individual and food changed as food enjoyment declined and as food preferences and aversions changed during treatment. This study presents relevant findings about the subject's relationship with food and reinforces the importance of understanding the influence of CT on perceptions associated with food consumption and HRQL to provide personalized care with better management of symptoms and adequate guidance on healthy eating habits.

## AUTHOR CONTRIBUTIONS

Marinho EC, Custódio ID, Paiva CE and Maia YC contributed equally to this work, they conceived and designed the experiments, performed the experiments, analyzed the data, wrote the paper and read and approved the final version of the manuscript. Ferreira IB and Crispim CA also contributed equally to this work, they analyzed the data, wrote the paper and read and approved the final version of the manuscript.

## Figures and Tables

**Table 1:** Clinical and therapeutic characterization of women with breast neoplasms (n=55).

Variable	Mean ± SD or n (%)
Age (years)	51.5±10.1
Weight (kg)	70.9±16.4
BMI (kg/m^2^)	28.3±6.4
Tumoral subtype	
Invasive ductal carcinoma	53 (96.4)
Invasive lobular carcinoma	2 (3.6)
Menopausal status	
Premenopausal	21 (38.2)
Postmenopausal	34 (61.8)
Type of surgery	
Conservative	24 (43.6)
Mastectomy	8 (14.6)
Did not undergo surgery (neoadjuvant)	23 (41.8)
Chemotherapy	
Adjuvant	32 (58.2)
Neoadjuvant	23 (41.8)
Chemotherapy protocol	
AC→ Docetaxel	33 (60.0)
AC→Paclitaxel	8 (14.6)
FAC	9 (16.4)
CMF	5 (9.1)

SD, Standard Deviation; BMI, Body Mass Index; AC, Adriamycin + Cyclophosphamide; FAC, 5-Fluoracyl + Adriamycin + Cyclophosphamide; CMF, Cyclophosphamide + Methotrexate + 5-Fluoracyl.

**Table 2:** Median of the hunger and meal enjoyment scales (n=23).

Variable	T0	T1	T2	*p*-value
	Median (p25-p75)	Median (p25-p75)	Median (p25-p75)	
Hunger	5.0 (2.0-5.0)	5.0 (3.0-8.0)	5.0 (2.0-6.0)	0.113
Meal enjoyment	9.0^a^ (7.0-10.0)	6.0^b^ (5.0-10.0)	9.0^a^ (8.0-10.0)	0.021*

T0, Principle cycle of chemotherapy; T1, Intermediate cycle of chemotherapy; T2, Last cycle of chemotherapy. ^a^ and ^b^, Horizontal medians followed by different letters differed statistically according to the post hoc test at the 5% probability level; *p<0.05. Of the 55 participants, 23 had meals in the institution at T0, T1 and T2, allowing a paired statistical test (n=23); (Friedman test and Tukey’s post hoc test).

**Table 3:** Description of the food preferences and aversions of women with breast neoplasms before and after chemotherapy (n=55).

Variable	T0		T2		*p*-value
	n	%	n	%	
Food preferences					
Meats in general	20	36.36	0	0	<0.0001***
Rice	16	29.09	0	0	<0.0001***
Vegetables	15	27.27	4	7.27	0.0100*
Fruits	11	20.00	6	10.90	0.2914
Legumes	10	18.18	0	0	0.0004***
Sweets in general	9	16.36	0	0	0.0027**
Bakery products	6	10.90	0	0	0.0271*
Coffee	4	7.27	0	0	0.1182
Tubers and roots	2	3.63	1	1.81	1.0000
Junk food	2	3.63	2	3.63	1.0000
Pasta	2	3.63	0	0	0.4954
Milk and dairy products	2	3.63	2	3.63	1.0000
Visceral	1	1.81	1	1.81	1.0000
Salty foods	1	1.81	3	5.45	0.6180
Acidic foods	0	0	2	3.63	0.4954
Coconut water	0	0	1	1.81	1.0000
Others	3	5.45	4	7.27	NA
Food aversions					
Vegetables	17	30.90	2	3.63	0.0002***
Fruits	10	18.18	7	12.72	0.5989
Meats in general	5	9.09	15	27.27	0.0244*
Milk and dairy products	4	7.27	2	3.63	0.6787
Beans	2	3.63	9	16.36	0.0524
Tubers and roots	2	3.63	0	0	0.4954
Sweets in general	2	3.63	5	9.09	0.4376
Rice	1	1.81	5	9.09	0.2057
Visceral	1	1.81	2	3.63	1.0000
Bakery products	1	1.81	3	5.45	0.6180
Junk food	1	1.81	2	3.63	1.0000
Coffee	0	0	8	14.54	0.0059**
Pasta	0	0	1	1.81	1.0000
Coconut water	0	0	5	9.09	0.0568
Garlic	0	0	2	3.63	0.4954
Soups	0	0	2	3.63	0.4954
Others	4	7.27	2	3.63	NA

Junk food: foods with high calorie content but with reduced levels of nutrients. Others: Foods mentioned only once including Asian cuisine, peanuts, mayonnaise, eggs, soybeans, seafood, grape juice, cod fish, isotonic foods, bitter foods, boiled foods, liquids, and crackers such as saltines. T0, First cycle of chemotherapy; T2, Last cycle of chemotherapy; NA, Not applicable; Parameters evaluated before the infusion of the first cycle of chemotherapy (T0) and 21 days after the last cycle (T2); *p<0.05; **p<0.01; ***p<0.001; (Fisher’s exact test).

**Table 4:** Quality of life of women with breast neoplasms undergoing chemotherapy (n=55).

Variable	T0		T1		T2		*p*-value
	Mean (SD)	Median (p25-p75)	Mean (DP)	Median (p25-p75)	Mean (DP)	Median (p25-p75)	
Overall health	78.6 (19.0)	83.3 (66.7-100)	77.1 (20.2)	75.0 (66.7-100)	74.4 (23.1)	75.0 (58.3-100)	0.618
Physical function	82.9 (19.9)	93.3^a^ (73.3-100)	78.1 (23.4)	86.7^a,b^ (60.0-100)	69.7 (25.0)	73.3^b^ (46.7-93.7)	0.002**
Role function	81.2 (27.0)	100^a^ (66.7-100)	65.4 (32.5)	66.7^a,b^ (33.3-100)	40.1 (40.6)	50.0^b^ (0.0-100)	<0.001***
Emotional function	63.9 (32.0)	75.0 (33.3-91.7)	62.4 (32.3)	75.0 (33.3-91.7)	58.3 (36.4)	66.7 (25.0-91.7)	0.233
Cognitive function	76.0 (27.9)	83.3^a^ (50.0-100)	68.8 (32.7)	83.3^a^ (50.0-100)	59.4 (36.5)	66.7^b^ (33.3-100)	0.014*
Social function	83.6 (23.7)	100 (66.7-100)	76.4 (28.3)	83.3 (50.0-100)	73.0 (31.3)	83.3 (50.0-100)	0.232
Fatigue	25.0 (27.1)	22.2^a^ (0.0-33.3)	30.5 (29.8)	22.2^a^ (0.0-55.6)	39.5 (32.0)	33.3^a^ (11.1-66.7)	0.002**
Nausea	4.5 (15.5)	0.0^a^ (0.0-0.0)	8.8 (20.0)	0.0^a,b^ (0.0-0.0)	12.7 (23.1)	0.0^b^ (0.0-16.7)	0.018*
Pain	27.6 (33.4)	16.7^a^ (0.0-50.0)	32.7 (37.7)	16.7^a^ (0.0-66.7)	46.7 (39.5)	50.0^b^ (0.0-100)	0.015*
Dyspnea	7.3 (22.8)	0.0 (0.0-0.0)	12.1 (26.7)	0.0 (0.0-0.0)	12.1 (23.4)	0.0 (0.0-33.3)	0.260
Insomnia	38.8 (41.9)	33.3 (0.0-66.7)	38.8 (39.4)	33.3 (0.0-66.7)	46.0 (42.8)	33.3 (0.0-100)	0.697
Loss of appetite	21.2 (37.6)	0.0 (0.0-33.3)	18.8 (33.8)	0.0 (0.0-33.3)	29.0 (39.5)	0.0 (0.0-33.3)	0.231
Constipation	21.2 (34.2)	0.0 (0.0-33.3)	26.0 (37.8)	0.0 (0.0-66.7)	21.2 (35.9)	0.0 (0.0-33.3)	0.623
Diarrhea	6.0 (18.2)	0.0 (0.0-0.0)	6.0 (19.2)	0.0 (0.0-0.0)	9.0 (26.0)	0.0 (0.0-0.0)	0.882
Financial difficulties	33.3 (38.5)	0.0 (33.3-66.7)	35.7 (37.9)	0.0 (33.3-66.7)	32.1 (40.0)	0.0 (0.0-66.7)	0.993
Body image	73.2 (32.1)	83.3^a^ (50.0-100)	72.1 (30.7)	83.3^a^ (50.0-100)	67.0 (34.6)	83.3^b^ (50.0-100)	0.046*
Sexual function	26.4 (25.4)	16.7 (0.0-50.0)	28.0 (26.1)	33.3 (0.0-50.0)	23.9 (24.6)	16.7 (0.0-41.7)	0.230
Sexual satisfaction	71.4 (19.1)	66.7 (66.7-83.3)	61.9 (30.3)	66.7 (33.3-100)	58.7 (34.8)	66.7 (33.3-100)	0.223
Future perspective	41.2 (40.5)	33.3 (0.0-66.7)	44.8 (42.2)	66.7 (0.0-100)	36.4 (39.7)	33.3 (0.0-66.7)	0.333
AEST	40.2 (22.3)	33.3 (23.8-52.4)	42.8 (22.8)	38.0 (23.8-57.1)	35.0 (22.1)	33.3 (19.0-47.6)	0.109
Symptoms of the arm	21.8 (21.2)	16.7 (8.3-33.3)	23.8 (25.4)	16.7 (0.0-33.3)	22.9 (26.1)	16.7 (0.0-33.3)	0.779
Symptoms of the breast	21.4 (21.2)	16.7 (8.3-33.3)	23.8 (25.4)	16.7 (0.0-33.3)	22.9 (26.1)	16.7 (0.0-33.3)	0.695
Loss of hair	59.2 (46.5)	66.7 (0.0-100)	62.9 (42.4)	100 (0.0-100)	44.4 (62.7)	0.0 (0.0-100)	0.368
Dry mouth^1^	43.6 (41.0)	33.3 (0.0-100)	50.9 (42.4)	33.3 (0.0-100)	41.8 (41.2)	33.3 (0.0-100)	0.214
Different flavor^1^	33.3 (42.5)	0.0 (0.0-66.7)	40.6 (39.4)	33.3 (0.0-66.7)	30.9 (38.4)	0.0 (0.0-66.7)	0.061

SD, Standard deviation; T0, Principle cycle of chemotherapy; T1, Intermediate cycle of chemotherapy; T2, Last cycle of chemotherapy; AEST, Adverse events of systemic treatment; ^1^Single Item; *p<0.05; **p<0.01; ***p<0.001. ^a^ and ^b^, Horizontal medians followed by different letters differed statistically according to the post hoc test at the 5% probability level (Friedman test and Tukey’s post hoc test).

**Table 5:** Correlation between differences in functional and overall quality of life domains (T2-T0) and differences in appetite scores for certain food groups (T2-T0) of women with breast neoplasms undergoing chemotherapy (n=55).

	Domains of quality of life (T2-T0)				
Appetite (T2-T0)	Overall health	Physical function	Role function	Emotional function	Social function
Foods rich in starch	0.099	-0.146	-0.182	-0.113	-0.298
Legumes	0.086	-0.538**	-0.235	-0.256	-0.333
Vegetables	-0.030	-0.461*	-0.241	-0.291	-0.203
Meats/eggs	0.004	-0.431*	-0.211	-0.226	-0.207
Soups/broths	0.344	-0.031	-0.328	-0.284	0.209
Fruits	-0.292	-0.091	-0.300	-0.277	-0.275
Juices	-0.446*	-0.388*	-0.266	-0.557**	-0.540**
Milk	-0.185	-0.152	-0.257	0.030	-0.219
Dairy products	-0.061	0.063	0.155	0.034	-0.146
Salty	0.100	-0.009	-0.184	0.037	0.074
Sweet	0.219	-0.115	0.060	0.141	0.362*
Acidic	-0.139	0.006	0.112	0.031	-0.108
Bitter	-0.003	-0.201	-0.072	-0.150	-0.303
Spicy	0.193	-0.098	0.024	0.299	0.133

T0, Principle cycle of chemotherapy; T2, Last cycle of chemotherapy; *p<0.05; **p<0.01; (Spearman correlation).

**Table 6:** Correlation between differences in some subscales of quality of life (T2-T0) and differences in appetite scores for certain food categories (T2-T0) of women with breast neoplasms undergoing chemotherapy (n=55).

	Quality of life scores (T2-T0)									
Appetite (T2-T0)	Fatigue	Nausea	Pain	Appetite	Constipation	Diarrhea	Body image	AEST	Dry mouth^1^	Different flavor^1^
Foods rich in starch	0.044	-0.061	0.380*	0.005	0.145	-0.091	0.166	-0.135	-0.092	-0.130
Legumes	0.202	-0.007	0.290	0.114	0.054	0.302	0.194	0.122	0.252	0.157
Vegetables	0.138	-0.154	0.358	0.071	-0.110	0.254	0.244	0.169	0.137	0.046
Meats/eggs	0.135	-0.020	0.089	-0.001	-0.048	0.294	0.093	0.163	0.270	0.106
Soups/broths	0.023	0.035	0.282	0.268	0.023	-0.007	0.154	0.283	0.180	0.278
Fruits	0.281	0.267	0.227	-0.081	0.118	-0.125	-0.082	-0.005	-0.235	-0.093
Juices	0.593**	0.313	0.440*	-0.011	0.331	-0.005	-0.274	-0.059	-0.122	-0.019
Milk	0.123	0.051	0.230	-0.083	0.023	-0.089	-0.281	-0.351	-0.315	-0.184
Dairy products	-0.065	0.076	-0.141	-0.196	-0.063	-0.047	0.017	-0.341	-0.120	-0.264
Savory	-0.001	0.209	-0.033	-0.143	-0.078	-0.229	0.045	-0.122	-0.137	-0.009
Sweet	-0.002	0.013	-0.186	0.224	-0.101	0.170	0.189	-0.211	-0.085	-0.152
Acidic	0.024	-0.071	0.052	0.257	0.146	-0.142	-0.160	-0.160	-0.231	0.093
Bitter	0.185	-0.019	0.366*	-0.043	0.113	-0.257	-0.068	-0.239	-0.300	-0.161
Spicy	-0.187	-0.183	-0.144	-0.031	-0.80	0.093	0.184	-0.053	0.009	0.019

T0, Principle cycle of chemotherapy; T2, Last cycle of chemotherapy; AEST, Adverse events of systemic treatment; ^1^Single Item; *p<0.05; **p<0.01; (Spearman correlation).
